# Medical Miscellanies

**Published:** 1817-01

**Authors:** 


### PART IV.

### MEDICAL MISCELLANIES.

### THE PORTE FEUILLE, No. I.

OR,

DEPOT FOR DETACHED FACTS AND INTERESTING PHÆNOMENA, OCCURRING IN THE PRACTICE OF THE FRIENDS OF THE MEDICO-CHIRURGICAL JOURNAL.

"Ore trahit quodcunque potest atque addit acervo."

### *Extract of a Letter from a Physician to Dr. Johnson.*

If the following hasty sketch of an *extraordinary disease of the thigh* be found adapted to the genius of your projected *Portfeuille*, it is much at your service: and I shall feel gratified by its insertion.

Some years since, a respectable tradesman, past the middle age, and long subject to indigestion and gout, accidentally fell; and struck the inferior posterior part of the right thigh, about the attachment of the short head of the *biceps flexor cruris muscle*, against a hard body. The pain, at first, was most exquisite; and never afterwards completely subsided. Soon, a hard, knotty substance, gradually increasing in size, was felt at the point upon which the injury had been inflicted. The whole thigh began to swell. Progression, at first painful, at length became impracticable. Every modification of local and constitutional treatment, employed to arrest the disease, availed nothing. On my first visit to the patient, I found him in bed. The disease had then subsisted for some months. The thigh was uniformly swollen from Poupart's ligament to the insertion of the great extensor crural muscles into the patella. The greatest circumference of the limb at least equalled that of the trunk to which it belonged. There was an evident sense of fluctuation throughout; but neither heat, discolouration, nor considerable pain on pressure. The leg was not at all implicated in the swelling; but paroxysms of most excruciating pain, alike abrupt in their onset and recession, were frequently felt at the extremity of the heel. Relief, during the access, could only be obtained from strong pressure of the part against a board, fixed for that purpose at the bottom of the bed. I ascribed this phænomenon to a remote sympathy of the extreme branches of the *nervus communicans tibiæ* with the disease in the thigh. Restlessness, anorexy, disordered bowels, fretfulness, emaciation, and debility, occupied the front rank in the assemblage of constitutional symptoms.

At that time, general compression of the thigh by resin plaster and rollers had been recommended by an eminent surgeon. Tried for some time, this plan served but to aggravate both the local and constitutional symptoms, without answering one good purpose. It was, therefore, determined, in consultation, to puncture the limb at the most prominent point of the swelling ; ascertain the nature of the contained fluid (supposed to be either blood or pus); and relieve the limb and system by partial evacuation of it. A small trocar was thrust in about the internal border of the *gracilis* muscle, at the distance of seven inches from its pelvic attachment: and, to the astonishment of all present, a stream of *beautifully transparent yellow fluid (having precisely the colour of fine cowslip wine), rushed from the orifice of the canula.* When a large chamber-pot full had been received, the current abruptly stopped ; and no manœuvre would restore it. The orifice was accurately closed by resin plaster ; and the limb, *little, if at all diminished in size by this operation,* was tightly encircled by a roller. The fluid, on cooling, was found to have *formed a firm consistent cake of jelly, capable of being turned out whole from the containing vessel.* I was not, at that time, sufficiently versed in practical chemistry to analyze the mass. Its physical characters were decidedly those of *pure animal gelatine.*

The wound healed by the first intention ; but the symptoms were not perceptibly mitigated. After the lapse of about a week, the operation was repeated ; and not more than a pint of fluid, exactly resembling the former, could be obtained. A third puncture, made some days afterward, yielded scarcely any ; and the orifice never completely closed. The patient, worn out, did not long survive the last operation.

Fearful of trespassing beyond the limits to which, I imagine, the articles introduced into your *Portfeuille* will be restricted, and unwilling to give a lame or hurried description of the anatomical survey which completes the history of this remarkable case, I shall postpone, till your next number, the account of a most deliberate dissection performed by myself, and noted at the time.

### *Case of Inflammatory Gout, terminating in Suppuration.*

A middle aged commercial gentleman, of temperate habits, but long and severely afflicted with wandering pains in the lower limbs, was, during the winter of 1815, seized with violent abdominal disorder; upon the decline of which, smart inflammation of the ball of the left great toe suddenly manifested itself. All the phænomena, local as well as general, announced the presence of *gout.*

A mercurial purgative, rest, and low regimen, were prescribed. Repeating my visit on the following day, I was surprized to see the ball of the toe covered by *a large, prominent vesicle, purple at the base, and fully distended with fluid.* The pain which, during the preceding night, had been most agonizing, decreased considerably on the formation of this deposit. Punctured with a needle, it yielded about half an ounce of thin, grey, horribly offensive pus. Poultices were now applied. In a few days, the cuticle separated ; and the concave ulcerated surface began to throw out healthy granulations. But the process of reparation, although uninterrupted, went on tardily, till pressure was employed ; whereby it was soon completed. Meantime, all the train of constitutional symptoms, except sluggishness of the bowels and debility, had taken leave. Bitters, alkalies, slight doses of blue pill, and nutritious diet, speedily removed these ; and restored all the animal and organic functions to a more than pristine vigour. The gentleman continues healthy; but feels the old pains of the limbs, whenever the atmosphere exhibits any sudden and unfavourable vicissitude.

### *Asthma alternating with Rheumatism and cured by spontaneous Hæmorrhage.*

A gentleman had long been afflicted with apparently asthmatic attacks, complicated, as was supposed, with some functional, if not organic derangement of the heart. He was occasionally seized with what he termed rheumatic gout, in different joints and parts of the extremities; and he observed, that during these seizures, the affection of the respiratory organs was considerably ameliorated. About three months ago, a violent hæmorrhage burst out from the nose; and notwithstanding every attempt which was made to restrain the discharge, some quarts of blood were lost. Since that time he has been much thinner in appearance, but the thoracic symptoms have entirely disappeared.

### *Series of Misfortunes in Parturition.*

A stout woman, 30 years of age, experienced a fright in the latter end of the ninth month of pregnancy. She was immediately seized with strong pains in the back, abdomen, and thighs, resembling those of parturition. These continued for seven days, without dilatation of the os uteri. At length, symptoms of real labour appeared, but the process was very slow; and on the second night, when the surgeon had retired, for a few hours, to rest, a midwife was secretly sent for, who promised to deliver the woman in a very short time. She immediately commenced, and prosecuted such a system of dilatation in the *os externum*, that when the surgeon returned next day, the labia pudendi were enormously swelled, inflamed, and apparently in a state of incipient sphacelus! The labour pains were entirely gone, and a suppression of urine had taken place. The head of the child was firmly impacted in the pelvis, and every effort to introduce the female catheter into the bladder failed. The bladder was felt like a small uterus at the lower part of the abdomen. A curved elastic gum catheter with the stillet inclosed was now introduced along the urethra, and the head of the child was pushed up off the pubis with all the strength of the other hand. It fortunately slipped into the bladder, and the urine was drawn off. As it was not certain that the child was dead, and as the woman's strength was still good, the head was not opened. Faint labour pains came on the next night; the head gradually advanced, and the child was protruded through a mass of œdema, swelling, and inflammation that almost closed up the external orifice of the vagina. The pain experienced during this expulsion was dreadful, and the woman's cries most affecting! The child was dead, and a gush of apparently liquid fæcal matter followed, which created a momentary apprehension that the woman's rectum had suffered; but it was soon ascertained to be the meconium of the fœtus mixed with the liq. amnii. The placenta was retained, and some hæmorrhage ensued. The hand was introduced, and met an hour glass contraction of the uterus. This was gradually overcome, and the placenta was found detached in the upper division of the uterus. It was extracted, and no hæmorrhage followed. In an hour, when the surgeon was taking leave, a most tremendous deluge of blood burst forth, and ran over the bedside on the floor. The windows were thrown open; the bed clothes removed; cold vinegar and water applied to the os externum and loins. The pulse was gone from the wrists; the face was cadaverous; the breathing short and sighing; the body covered with a cold clammy sweat. The hæmorrhage stopped; but for two or three hours it was doubtful whether the vital spark would re-kindle or become quite extinct. Very gentle cordials were administered; slight pulsations returned to the wrists; the respiration became less hurried, and deeper; in short, the system rallied from the direful shock.

On the second day after delivery, to the symptoms of inflammation and irritation in the vagina were added those of uterine, if not peritoneal inflammation, with low fever, restlessness, and great tenderness in the hypogastric region. Purgatives, calomel and opium every four hours, and fomentations removed these symptoms in three days.

Then a new train of disasters was developed. The whole internal surface of the vagina had sloughed, and what was worse, the neck of the bladder had given way; for the urine as it was secreted percolated through the vagina over the raw surfaces causing the most indescribable agonies! Lotions with opium and starch were constantly thrown up, and gave some alleviation, but the misery continued for six days; when the natural surface of the vagina got into a better state, and the constant drain of urine from the bladder gave much less pain.

She sometimes advanced, sometimes retreated, for several weeks; the sufferings from the passage of the water over the ulcerated surfaces being very great occasionally. There was not any appearance of being able to retain her water at the end of the fifth week; but she sits up an hour or two every day. Some fever is always present. At the end of the sixth week, little complaint was made of the pain in passing water; but an obscure hardness was felt in the right hypogastrium, with great pain, constant fever, loss of appetite, and indications of hectic.

The linimentum hydragyri was rubbed on that side of the abdomen till the mouth became sore, keeping the bowels open. The hardness and swelling disappeared; the febrile symptoms ceased; the appetite returned. In four weeks, the general health was so far re-established, that she left her bed, and could walk about. The incontinence of urine gradually went off, and in short, she perfectly recovered.

### *Debility removed by Bleeding.*

A young gentleman, 15 years of age, came home from an academy with symptoms indicative of typhus mitior. The debility, whether real or apparent, was so great, that he could scarcely walk up stairs, though he had been but three days ill. The tongue was loaded; the voice faultering; the head heavy; the skin rather hot; he had chilly fits alternating with flushes, and could not sleep. The pulse was neither hard nor soft, but was quicker than natural. On opening a vein in the arm, he fainted before six ounces were abstracted. After this he did not complain of the chilliness; but by the end of the fourth day, the debility was such, that he could not turn himself in his bed; and having taken some opening medicine, he complained of total inability to put the abdominal muscles in action for the purpose of expelling the fæces. He lay in any position in which he was placed; and had delirium. The carotids throbbed; and the eyes were watery. Notwithstanding the most urgent remonstrances of the friends, it was insisted on that leeches should be applied to the temples. They were applied; and during the night, sixteen napkins were completely drenched with blood. The debility diminished in exact proportion as the blood flowed; and next morning he could sit up in the bed, help himself to drink, and converse cheerfully with his friends. Previous to the abstraction of blood by leeches, he could not clothe his ideas in language but with much difficulty, having to search, as it were, for every word; but after the discharge, the first remark he made in the morning to his medical attendant was, that his memory was restored; for he could name any thing, or any body with facility. The calomel, which had been lying in his bowels all the preceding days, now acted briskly; and he found no difficulty in applying the force of the diaphragm and abdominal muscles towards the expulsion of the fæces.

The subsequent night he slept well, and was very much refreshed; nevertheless, the fever maintained an easy and quiet pace without manifesting a determination to any viscus.

What would have been the effects of a Brunonian mode of treatment, when debility was so *apparent* at the commencement of this fever?

### MISCELLANEOUS.

Quicquid agunt Homines.

The following letter has reached us without the authentication of any real name. As it relates to a subject of the greatest importance to a numerous class of the profession, we have given it insertion. We forbear to express any opinion on the decision of the Court of Examiners, but our pages will be open to such of our Correspondents on the other side as may be inclined to vindicate their conduct by fair and temperatearguments. *Audi alteram partem* is a maxim, the propriety and justice of which, we willingly acknowledge. EDITORS.

*To the Editors of the Medico-Chirurgical Journal & Review.*

GENTLEMEN,

The subject of the following letter involves a question, which, with its solution, is of such vital importance to every Apothecary, that it cannot fail of being of the highest interest to every member of that branch of the medical profession.

This Document is not, I believe, in very general circulation; though of course it can be no secret, when it has been pretty generally and warmly discussed among the Members of the Courts of Assistants and Examiners. It is copied verbatim, excepting that the names of the parties, are omitted. But they who read the letter will find little difficulty to fix on the writer of it.

If doubt exists of its authenticity, that may be easily solved, by inquiring of any of the members of either Court; many of whom will, perhaps, exert themselves to suppress this exposure.

I rely on your independent character as Editors of a Public Journal to give it the earliest publicity.

I am, Gentlemen,

Your obedient servant,

AN APOTHECARY.

"GENTLEMEN, October 2, 1816.

"Having been appointed by you a Member of the Court of Examiners of the Society of Apothecaries, and a circumstance having occurred in the execution of that duty, which renders an appeal to the Court of Assistants imperative upon me, I beg leave to state the case to your consideration and decision.

"A candidate of the name of A. B——, applied on the 12th ultimo, to the Court of Examiners, to be examined for a Certificate to practice as an Apothecary.

"He stated that he had been bound an Apprentice, as a *Citizen and Ironmonger*, in the year 1807, for seven years, to a Mr. C. D——, Chemist and Druggist, of Street, London; that his Indenture was in the Chamberlain's Office; but his Master had given him the following Certificate, which he produced.

"I hereby notify to whom it may concern, that A. B——,
"was bound apprentice to me on the 3d day of November 1807,
"for the term of seven years; that during the said period he
"conducted himself to my satisfaction; that he was in the habit
"of dispensing medicines, and making preparations of pharmacy,
"under my directions, and that he is of good moral character.

"Signed (C. D.)"

"London, June 18, 1816."

"Half the Members of the Court, including myself, objected to Mr. B—— being admitted to examination; upon the ground that he had not served an apprenticeship to an Apothecary, as the act directs; and, therefore, could not claim under the act to be examined.

"The casting vote decided, that he should be admitted to examination. I then offered a formal and written dissent, founding my objection on its being an illegal proceeding.

"A proposal to postpone deciding the question of examination for a week was also rejected.

"As I considered the proceedings as contrary to law as it was to policy, I refused putting my signature to a certificate, granted under such circumstances; and the reasons that influenced my determination are:

"1. That the candidate had served his apprenticeship to a Chemist and Druggist, and not to an Apothecary.

"2. That the master's certificate did not set forth or assume, that he was an Apothecary, but a mere dispenser of medicines.

"3. That a dispensing Chemist and Druggist, who neither before nor since the passing of the act, ever called himself, or wrote upon his house, *Apothecary*, is not an Apothecary, nor can his apprentice claim as such.

"4. Because whatever doubts might have prevailed formerly, as to who were or who were not Apothecaries, none can exist now; since the Committee of the House of Commons would not grant the prayer of the petition for the late act, until evidence had been examined, that Apothecaries had from time immemorial visited and prescribed for the sick; and unless satisfactory proofs of that fact had been adduced before them, the act, which expressly enjoins the Court of Examiners to examine all Apothecaries touching '*their skill and abilities in the science and practice of medicine*,' [See Section 14.] would never have passed.

"5. That the said master is not an Apothecary, is also clear, according to the usage of the Society; for his shop is exempt by former custom, as well as by the act itself, from the inspection of the Society's Examiners; nor has it ever been examined by the College of Physicians, although it has remained in its present state for thirty years.

"6. That no apprentice, who had served this master, or any other hemist and Druggist. would be admitted to purchase his freedom of the Society, which, if I am rightly informed, always require indentures of apprenticeship to a member of the Society or to a regular Apothecary.

"7. The Chemists and Druggists opposed the bill, and procured the introduction of a special clause [See Section 28] to free them from being considered Apothecaries, and being subjected to the provisions of the act.

"8. That the Court of Assistants have themselves sanctioned the opinion, that dispensing Druggists and Chemists were not Apothecaries.

"9. That the Court of Examiners have themselves also decided, that persons serving an apprenticeship to Chemists and Druggists solely, have no right to be examined or to practise as Apothecaries.

"Lastly, If it be established, that dispensing Chemists and Druggists are *legal* Apothecaries, the Court of Assistants cannot by virtue of any bye law refuse any Chemist or Druggist admission into the Society, after having received a certificate as a qualified Apothecary. Thus a precedent may be introduced, that may prove subversive of the Society itself as a body of Apothecaries.

"These, Gentlemen, are not merely my arguments; they embrace the opinions of the best legal authority I have been able to consult. But as the legal opinions to which I allude, could not be received, as they did not come through the official and regular channel, I submitted a motion at the last meeting of the Court of Examiners, that the Court of Assistants should be requested to take Council's opinion on a subject so delicate and important, in order that the minds of all the Examiners might be correctly informed, and the consciences of the majority of them be satisfied, whether they are acting right or wrong. But this question was also negatived by a casting vote only.

"Failing, therefore, in my attempt to lay this case before your Court in the more consistent way, my only resource is, to make a personal appeal for advice to guide my future conduct on any similar occasion.

"If the law be otherwise than I have been taught, and interpret it, however much I may lament the defect in the act, it is my duty to submit and conform to its enactments; but having at present a quite opposite impression, I feel it equally my duty as a member of the Court of Examiners, until instructed by legal authority to the contrary, to firmly resist, by every fair means, all attempts to admit candidates having served dispensing Chemists and Druggists only, to an examination. Neither will I sign the certificate of any such candidate.

"I therefore most respectfully solicit from the Court of Assistants an early intimation of their pleasures on the subject now proposed to them.

"I have the honour to be

"Gentlemen,

"Your obedient humble servant,

——————"

"To the Master, Wardens, and Court of Assistants of the Society of Apothecaries."

*At a meeting of the* MEDICAL SOCIETY, *held at their House in Bolt Court, Fleet Street, on the 9th of December last*, Mr. STEVENSON *presented for inspection his new Instruments adapted to diseases of the Eye and Ear.*—The first of these which was much wanted in surgical practice, is a speculum auris, which serves the compound use of assisting in opening the outer part of the meatus auditorius externus, and of enabling the practitioner to extract polypi, or to remove any extraneous substances introduced into the tube.

A very neat scarificator was next exhibited, constructed so as to answer the double purpose of dividing, with equal success, the vessels of the inflamed and turgid membrane of the auditory passage, as well as those of the palpebraic conjunctiva. This and the former instrument were particularly admired, and approved of by all present.

The mechanism and use of a cataract knife, of a new form, was described with sufficient accuracy to impress us with the belief, that it is a great improvement upon those of Baron Wenzel, and Beer of Vienna, which are in general use, by enabling the surgeon to make a better and more convenient incision through the cornea for the extraction of the opake lens.

The evacuation of the aqueous humour in certain descriptions of ocular complaints being found highly beneficial, an instrument was shewn, apparently well adapted for that purpose, as well as for removing, by the aid of a grooved silver conductor, pus, blood, or even the broken fragments of a cataract from the anterior chamber of the eye. These, which, as far as we recollect, were the whole of the instruments submitted to the examination of the Society, bespeak great ingenuity in the inventor, as well as a thorough knowledge of the principles and practice of that branch of surgery, to which Mr. Stevenson, in a manner no less honourably than usefully, devotes his time and talents.

---

*Hydatids voided by the Urethra.*—The subject of this case, a general officer, suffered from urethral stricture, consequent on an old gonorrhœa. A greenish fluid incessantly oozed from the canal. The urine was voided with difficulty, and deposited a copious puriform and fœtid sediment: and the perinæum and scrotum were covered with an herpetic eruption which itched violently. Baths, diluent liquids, and mucilaginous injections, were prescribed in vain. The patient, afterwards, tried partial immersion of the body in a solution of corrosive sublimate (oxymuriate of quicksilver) the strength of which was regularly incresead. A small portion of the mineral preparation was likewise dissolved in the injections. The patient, in addition to these, employed a plaster bougie, and drank the waters of Contrexeville. Nevertheless, his condition became more alarming. The appetite was irregular. He grew peevish and emaciated. A pain, at first obtuse, but soon augmenting to an intolerable degree, attacked the loins; and a slight swelling appeared in the region of the left kidney. A few days after the accession of these symptoms, the patient discharged from the urethra, first one hydatid, and afterwards others. He was then directed to drink Seltzer water. Seventeen of these hydatids having been successively voided, the functions of the urinary organs were gradually re-established; and the health of the patient was, at length, perfectly restored.—*Aulagnier, Recueil périodique de la Société de Médecine de Paris, Mai et Juin,* 1816.

The dissection of the Hottentot Venus, which was lately performed at Paris by Professor Cuvier, has afforded an opportunity of ascertaining the truth of the description given, by various travellers into the interior of Africa, of a kind of natural apron, which is said to cover the parts of generation of the females of several tribes of the natives of that part of the world. This peculiarity seems to belong more particularly to a people denominated by the Dutch inhabitants of the Cape, *Boschismans,* who are of ferocious manners, living by rapine and plunder, and whose persons bear evident marks of the sufferings and ill-usage they undergo. The females of this tribe are not only distinguished by the apron in question, but also by an extraordinary enlargement of the buttocks; and there is no doubt but that, as the individual now dissected had this characteristic mark, she was of the race named by travellers *Houzouanas.*

When examined during life, this woman contrived, although completely naked, to conceal from the view of observers the peculiar conformation of the genitals, which was only demonstrated by dissection. There existed a kind of double excrescence, formed by the union of the nymphæ, in length about two inches, anteriorly and superiorly by an extension of the skin, which served as a prepuce to the clitoris. This conformation had not any resemblance to an apron, but consisted merely of two triangular membranes united anteriorly at their base. As to the fleshy protuberance of the buttocks, this did not consist of muscular substance, as had been supposed, but was entirely cellular, crossed in all directions by a very dense fibrous texture, which gave it a great degree of elasticity. The bony structure of the head of this woman exhibited the peculiarities of the Negro; the nose was much flattened, while the lower part of the face advanced very forward, and the features were characteristic of the Mongolian race; that is, the upper part of the face was very broad, with a projection of the cheek-bones. No other head, examined by M. Cuvier, exhibited the first of these characters to so great a degree, consequently ever approached so nearly the structure of the inferior animals. M. Cuvier has compared the heads of a great number of individuals of different races, with a view of pointing out their resemblance or difference: the heads of all the *mummies,* whatever be their antiquity, indicate the Egyptians to have been of Caucasian origin It is the same with the *Guanches* of the Canary isles, who, as well as the Egyptians, were in the habit of converting their corpses into mummies.—*Gazette de Santé, Nov.* 1816.

Dr. Burrows of Gower Street, is preparing for the press, a volume of "Commentaries on Mental Derangement."

Dr. Johnson, one of the Editors of this Journal, has a vacancy for a Pupil, who will be instructed in the various branches of the profession, and treated as one of the family. Two winters out of the five will be allowed for Lectures, Hospitals, &c. and an energetic impulse will be given to the pupil's studies.—Premium, including board, £. 300.

☞ None but those of respectable Connections need apply.

Middlesex Hospital.—Dr. P. M. Latham, and Dr. Southey, will commence a second Course of Lectures, on the Practice of Physic, at this Hospital, on Monday, the 3d of February next. Particulars may be known by applying to Mr. Heath.

Theatre of Anatomy.—Mr. John Taunton will commence his Winter Course of Lectures on Anatomy, Physiology, Pathology, and Surgery, on Saturday, January 18th, 1817, at eight o'clock in the evening precisely; they will be continued every Tuesday, Thursday, and Saturday, at the same hour. Particulars may be had on applying to Mr. Taunton, 87, Hatton Garden.

Theatre of Midwifery, 68, Berner's Street.—Dr. Clough, Physician Accoucheur to the Endeavour Society; to the St. Mary le-bone General Dispensary; and to the Benevolent Institution for the delivery of poor Women at their own habitations, &c. &c. commences his Winter Course of Lectures on the above science, &c. on Monday morning, the 6th of January, at half past ten.

Deaths—In Princes Street, Cavendish Square, Wm. Royston, Esq. formerly one of the Editors of the Medical and Physical Journal.—At Heavitree, near Exeter, Wm. Cooper, Esq. formerly Surgeon to Guy's Hospital.—

